# Expression and Localization of a Testis‐Specific Heat Shock Protein (CfHSP70‐2) During Spermatogenesis in the Spruce Budworm, *Choristoneura fumiferana* (Clem.)

**DOI:** 10.1002/arch.70192

**Published:** 2026-08-25

**Authors:** Guoxing Quan, Hannah Soares

**Affiliations:** ^1^ Natural Resources Canada, Canadian Forest Service, Great Lakes Forestry Centre Marie Ontario Canada

**Keywords:** heat shock protein, spermatogenesis, spruce budworm, testis

## Abstract

Heat shock proteins (HSPs) are essential across several insect species for adaptation to environmental stressors. Most pest insects experience fertilization concurrently with summer heat extremes, which mostly affect male testis development and spermatogenesis; although the molecular mechanisms involved are unclear. Several HSPs are exclusive to the testis in several insects, but their functional roles remain unclear. In this study, we identified a testis‐specific ATP‐dependent HSP, *CfHSP70‐2* in the spruce budworm (SBW), *Choristoneura fumiferana*. Reverse transcript‐qualitative polymerase chain reaction and western blotting were performed to detect the tissue‐and developmental stage‐specific expression of CfHSP70‐2. In addition, the effects of various stressors on CfHSP70‐2 expression in the testes were examined. Importantly, CfHSP70‐2 was first detected in the late pupal stages and peaked in the adult stages. Heating, starvation, or chemical HSP activators did not stimulate CfHSP70‐2 expression. Immunohistochemical staining revealed that CfHSP70‐2 was primarily found in round spermatids at the late pupal and adult stages. The potential function role of CfHSP70‐2 in spermatogenesis in SBW is discussed.

## Summary

- *Choristoneura fumiferana* expresses heat shock protein CfHSP70‐2 only in the testis.
- Late pupal and adult stages exhibit high expression of CfHSP70‐2 mRNA and protein.
- Heat shock, starvation, or chemical HSP activators could not induce CfHSP70‐2.
- CfHSP70‐2 is primarily found in round spermatids during spermatogenesis.

## Graphical abstract

This study discovered CfHSP70‐2, a heat shock protein expressed in the testis of a forest lepidopteran pest spruce budworm, *Choristoneura fumiferana*, during late pupal and adult stages primarily found in round spermatids during spermatogenesis. Heat shock, starvation, and chemical heat shock protein activators were unable to alter expression.

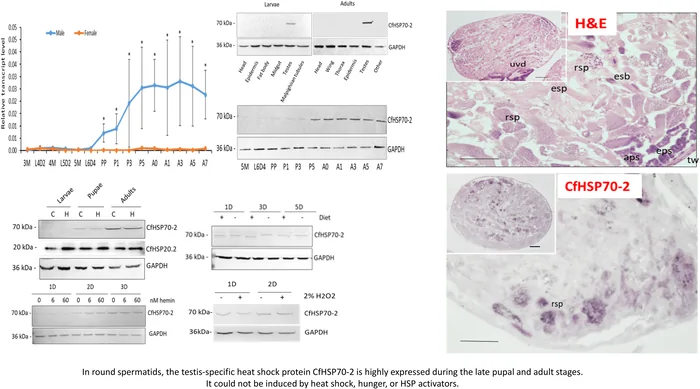

## 1 Introduction

The majority of insect pests are ectotherms, and their distribution and population dynamics are affected by warming climates (Colinet et al. 2015). Temperature has sex‐dependent effects on heat‐ and/or cold‐tolerance, longevity, and fertility in several insect species (Fischer and Fiedler 2000; Rako and Hoffmann 2006; Boersma et al. 2018; Saxon et al. 2018; Parratt et al. 2021; Sales et al. 2021). Late‐instar larvae, pupae, and adults experience peak summer temperatures in the Northern Hemisphere (Miller 1975; Fleming and Volney 1995). Globally, extreme temperature events are increasing in frequency, intensity, and duration due to climate change (Colinet et al. 2015).

Many insect pests experience heat stress‐induced fertilization, which mostly affects male spermatogenesis (David et al. 2005; Sales et al. 2018; Sales et al. 2021). In Diptera, *Drosophila* spermatogenesis has been predicted to be more sensitive to thermal stress than oogenesis (David et al. 2005; Canal Domenech and Fricke 2023; Ørsted et al. 2024). In Coleoptera, heatwaves reduce the number and viability of red flour beetle sperm (Sales et al. 2018). In Lepidoptera, exposure of silkworm pupae to heat shock causes sterility (Sahara and Kawamura 2002). However, the molecular mechanisms underlying heat stress‐induced sterility remain unclear.

Heat shock proteins (HSPs) are ubiquitous and conserved in cells of all living organisms, including prokaryotes and eukaryotes, and act as molecular chaperones. HSPs associate with co‐chaperones and target to form molecular networks that promote the correct folding of nascent and stress‐accumulated misfolded proteins preventing irreversible protein aggregation and/or promoting the degradation of misfolded or aggregated proteins (Sørensen et al. 2003). Over the years, several HSP genes have been isolated and studied in insects. Environmental stresses, such as heat, cold, starvation, drought, dehydration, ultraviolet radiation, and chemical pesticides, can upregulate or downregulate the expression of HSPs and enhance stress tolerance or resistance (Zhao and Jones 2012; King and MacRae 2015). The majority of HSPs in insects are not stress‐inducible and are produced in a life stage‐ and/or tissue‐specific manner; however, their in vivo functions and the mechanisms of action remain unclear (King and MacRae 2015; Zhang et al. 2022). In addition, the roles of these HSPs, particularly in the testis development and spermatogenesis under non‐stress conditions, are nearly unknown.

The spruce budworm (SBW), *Choristoneura fumiferana*, a lepidopteran forestry pest, is widely distributed in spruce and balsam fir forests across North America (Régnière et al. 2012; Frago and Bauce 2014). Natural factors such as temperature extremes, drought, and food shortages diminish SBW outbreaks and their geographical distribution (Candau and Fleming 2005; Frago and Bauce 2014). Our previous study identified an ATP‐independent small HSP CfHSP20.2, which may be involved in gonad development and contribute to heat tolerance (Quan et al. 2023). In another previous study we identified seven ATP‐dependent *HSP70s* in SBW (Quan et. 2020), and further investigation found that two HSP70s were highly expressed in the males at transcript levels (S1). CfHSP70‐2, one of the two male‐specific HSPs, was successful in creating an antiserum, allowing us to perform further research. As a first step toward understanding the molecular basis of heat stress‐induced sterility, and the potential roles of HSP in the testis development and spermatogenesis, we investigated its mRNA and protein levels during testis development, localization in the testis, and responses to stress. In addition, we explored the potential roles of CfHSP70‐2 during testis development and spermatogenesis.

## 2 Materials and Methods

### 2.1 Experimental Insects

SBWs, originating from Northern Ontario, were provided by Insect Production Services, Great Lakes Forestry Centre, Sault Ste. Marie, Canada. SBWs were reared as larvae on an artificial diet modified from McMorran (1965) and maintained under controlled conditions (temperature, 22°C–23°C; relative humidity, 55%–60%; 16:8 light: dark regime). Males and females were separated during larval development for subsequent analysis.

### 2.2 Heat Shock, Starvation, Hemin, and H_2_O_2_ Injections

Briefly, 4th‐day 6th‐instar larvae (L6D4), 3rd‐ pupae (P3), or newly emerged adults were placed in Petri dishes and incubated at 37°C for 1 h and then maintained under controlled conditions (temperature, 22°C–23°C) to recover for 1 h. Subjects from the same batch of insects at the same stage of life that did not undergo heat treatment were used as controls. To investigate the effects of starvation on CfHSP70‐2 expression, 1st‐day 6th‐instar larvae (L6D1) were placed in empty Petri dishes and maintained under standard rearing conditions. Larvae reared under standard conditions, but given a regular diet were used as controls. Testes were dissected on days 1, 3, and 5 for subsequent analysis. Each experimental group had 15–20 testes and 4 independent replicates. Male 1st‐papae (P1) were injected with hemin (final concentration of 6 and 60 µM) or 2% H_2_O_2_ (Millipore Sigma Canada Ltd.). Pupae injected with phosphate‐buffered saline (Millipore Sigma Canada Ltd.) were used as controls. After injection, the pupae were maintained at 23°C and 60% relative humidity. Testes were dissected at specific time points for the protein isolation and western blotting analysis.

### 2.3 RNA Isolation and Reverse Transcription‐Quantitative Polymerase Chain Reaction (RT‐qPCR)

Total RNA isolation, cDNA generation, RT‐qPCR, and detection were performed as described by Quan et al. (2018). *CfHSP70‐2* mRNA expression was qualified using at least four biological replicates. Finally, the relative expression of the target gene was estimated using the 2^‐∆∆CT^ method (Livak and Schmittgen 2001) and normalized to that of the housekeeping gene, translation elongation factor‐1α (Tef‐1α), as previously described (Quan et al. 2018).

### 2.4 Western Blot Analysis

To extract proteins for subsequent analysis, entire insects or their dissected tissues were homogenized in Radio‐Immunoprecipitation Assay (RIPA) buffer (10 mM Tris‐HCl, pH 8.0, 140 mM NaCl, 1 mM EDTA, 0.5 mM EGTA, 1% Triton X‐100, 0.1% Sodium Deoxycholate, 0.1% SDS) containing a protease inhibitor cocktail (Millipore Sigma Canada Ltd.), incubated on ice for 30 min, and centrifuged at 20,000 × g for 10 min at 4°C. Protein concentration was determined using the Qubit Protein BR Assay Kit (Thermo Fisher Scientific Inc.) and QFX Fluorometer (DeNovix) with bovine serum albumin used as the protein standard. Protein samples were mixed with 4x loading dye (1x loading dye is 0.625 M Tris‐HCl, pH 6.8, 40% glycerol, 4% SDS, 2% 2‐mercaptoethanol, and 0.01% bromophenol blue) and denatured for 10 min at 70°C before loading. Protein samples containing 20 µg of protein each were size‐fractionated on a 4%–20% sodium dodecyl sulfite polyacrylamide gel (GenScript USA Inc.) and transferred to a polyvinylidene difluoride (PVDF) membrane using the iBlot Dry Blotting System (Thermo Fisher Scientific Inc.).

A rabbit polyclonal antiserum was generated using the synthetic peptide, PSCARPASPGGPRVQE, as the antigen (Biomatik, Canada Co.). This peptide corresponds to the C‐terminal amino acids 617–632, which are unique to all known HSP70 proteins in SBW (Quan et al. 2020). The antiserum was diluted 1:1,000 in blocking buffer (4% skim milk powder in washing buffer) to obtain a final concentration of 0.5 µg/mL The specificity of the CfHSP70‐2 antibody was determined through western blotting analysis using pre‐bleed as the first antiserum, where no bands were detected (data not shown), and it recognized the recombined CfHSP70‐2 protein expressed in baculovirus‐infected SF21 cells (Data not shown). All results indicated that the antiserum was specific for the CfHSP70‐2 protein. An anti‐GAPDH antibody (Sigma‐Aldrich Canada Co.) was used as the loading control (1:2,000). Goat anti‐rabbit IgG conjugated with horseradish peroxidase was used as the secondary antibody (Bio‐Rad Laboratories (Canada) Ltd.). After the first and second antibody incubation, membranes were washed in 1x TBST (20 mM Tris‐HCl (pH7.6), 150 mM NaCl, and 0.2% Tween‐20) four times for 5 min each with gentle rocking. Signals were visualized using the ImmPACT DAB EqV Substrate Kit (Vector lab Inc. USA).

### 2.5 Paraffin Sectioning And, Hematoxylin and Eosin Staining

Testes of larvae, pupae, and adults were dissected and fixed overnight at 4°C with 10% neutral‐ buffered formalin solution (Sigma‐Aldrich, Canada). After fixation, the testes were dehydrated in increasing concentrations of ethanol (70%, 90%, and 100%), respectively, cleared three times using xylenes, embedded in paraffin overnight, and cut into 7‐μm‐thick sections using a Lipshaw 45 Rotary Microtome. Thereafter, the sections were stained using the hematoxylin and eosin stain kit (Vector laboratories Inc.) to visualize testicular morphology. Finally, the stained sections were photographed using an Olympus BX51 microscope and a Nikon DS‐Fi3 camera. Apyrene spermatocytes have smaller, denser nuclei and cytoplasm than eupyrene spermatocytes (Friedländer et al. 2005).

### 2.6 Immunolocalization of CfHSP70‐2

Briefly, cross‐sections of the testes were prepared using the same method described above. Thereafter, the unwaxed sections were treated with antiFix™ universal antigen retrieval buffer (Bioyium Inc.) according to the manufacturer's instructions. After rinsing in 4% skim milk powder in TBST buffer, the slides were stained with a rabbit polyclonal antibody CfHSP70‐2 antibody (200x dilution) at 4°C overnight. Thereafter, the sections were treated with a peroxidase suppressor (Thermo Fisher Scientific, Canada) to inhibit endogenous peroxidase activity, and incubated with a goat anti‐rabbit alkaline phosphatase (AP)‐conjugated serum (Bio‐rad Laboratories Ltd.). Finally, colorimetric immunodetection was performed using the BCIP/NBT (5‐bromo‐4‐chloro‐3‐indolyl phosphate/nitroblue tetrazolium) AP Substrate Kit (Vector Laboratories Inc. USA). The specificity of the reactions was determined by omitting the first antiserum as a control (S2).

### 2.7 Statistical Analysis

All data are presented as mean ± standard deviation (SD). Data obtained from at least four independent biological samples were evaluated using one‐way analysis of variance (ANOVA) followed by a Tukey's post hoc test. Statistical significance was set at *p* < 0.05. All statistical analyses were performed using Microsoft 365 excel.

## 3 Results

### 3.1 Stage‐ and Tissue‐Specific Expression of *CfHSP70‐2*

**Figure 1:**
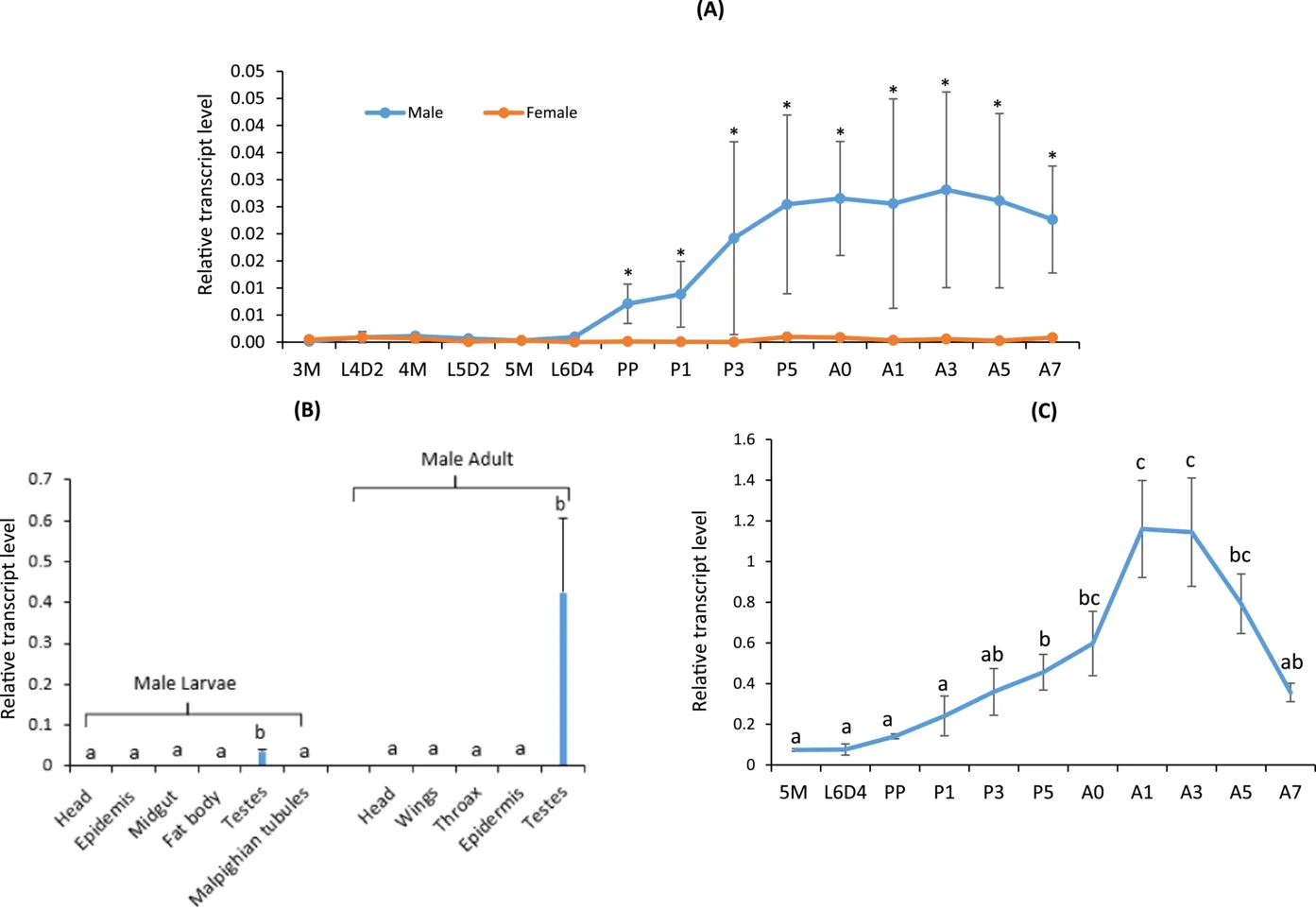
Stage‐ and tissue‐specific expression of *CfHSP70‐2* transcript. Relative *CfHSP70‐2* mRNA levels in various tissues at different developmental stages are presented as means ± standard deviation (SD) and expressed as apparent levels of Tef‐1α expression determined using RT‐qPCR. (A) Stage‐specific expression of the transcript in males and females at various developmental stages. (B) Tissue‐specific expression of *CfHSP70‐2* in L6D4 larvae and adults. (C) *CfHSP70‐2* mRNA expression in testes at various developmental stages. 3 M: molting 3rd; L4D2: 2nd day 4th‐instar larvae. 4 M: 4th‐molting instar larvae; L5D2: 2nd‐day 5th‐instar larvae; 5 M: molting 5th instar larvae; L6D4: 4th‐day 6th‐instar larvae; PP: pre‐pupae; P1–P5: pupae 1 to 5 days following pupation; A0: newly emerged adults; A1–7: adults 1 to 7 days following eclosion. Asterisks and different letters above the bars indicate the significant difference in expression levels as determined by t‐test.

RT‐qPCR was performed to examine the expression profiles of *CfHSP70‐2* in male and female larvae, pupae, and adults. *CfHSP70‐2* mRNA expression was extremely low in male and female larvae. In females, *CfHSP70‐2* mRNA expression remained consistently low throughout the pupal and adult stages. In contrast, *CfHSP70‐2* mRNA expression levels increased in pupae in males, reaching its highest level in the early adult stage and then slightly decreasing in day‐5 and ‐7 adults. Notably, *CfHSP70‐2* mRNA expression was higher in male adults than in female adults by approximately 44‐fold (Figure 1A), suggesting that *CfHSP70‐2* is expressed specifically in males. To investigate the tissue‐specific distributions of *CfHSP70‐2*, we examined its expression in various tissues in male larvae and adults using RT‐qPCR. *CfHSP70‐2* mRNA expression was detected only in the testes of larvae and adults (Figure 1B). Importantly, *CfHSP70‐2* mRNA expression in the testes of larvae and adults were 182‐fold and 472‐fold higher, respectively, than that in the whole insects. Collectively, these results indicate that *CfHSP702‐2* expression is testis‐specific, with expression in other tissues in males and females remaining below the detectable threshold (data not shown for females). In particular, *CfHSP70‐2* expression remained low in larvae and gradually increased in pupae, peaking in adults during their first 3 days of life. Moreover, the highest expression levels were observed in day ‐1 and ‐3 adults, with approximately a 15‐fold higher expression than that in the testes of fifth molting larvae (Figure 1C). During the adult stage, *CfHSP70‐2* expression gradually declined, reaching approximately 30% of the levels observed on day ‐1, in day‐5‐ and ‐7 adults.

### 3.2 Stage‐ and Tissue‐Specific Expression of CfHSP70‐2 Protein

**Figure 2:**
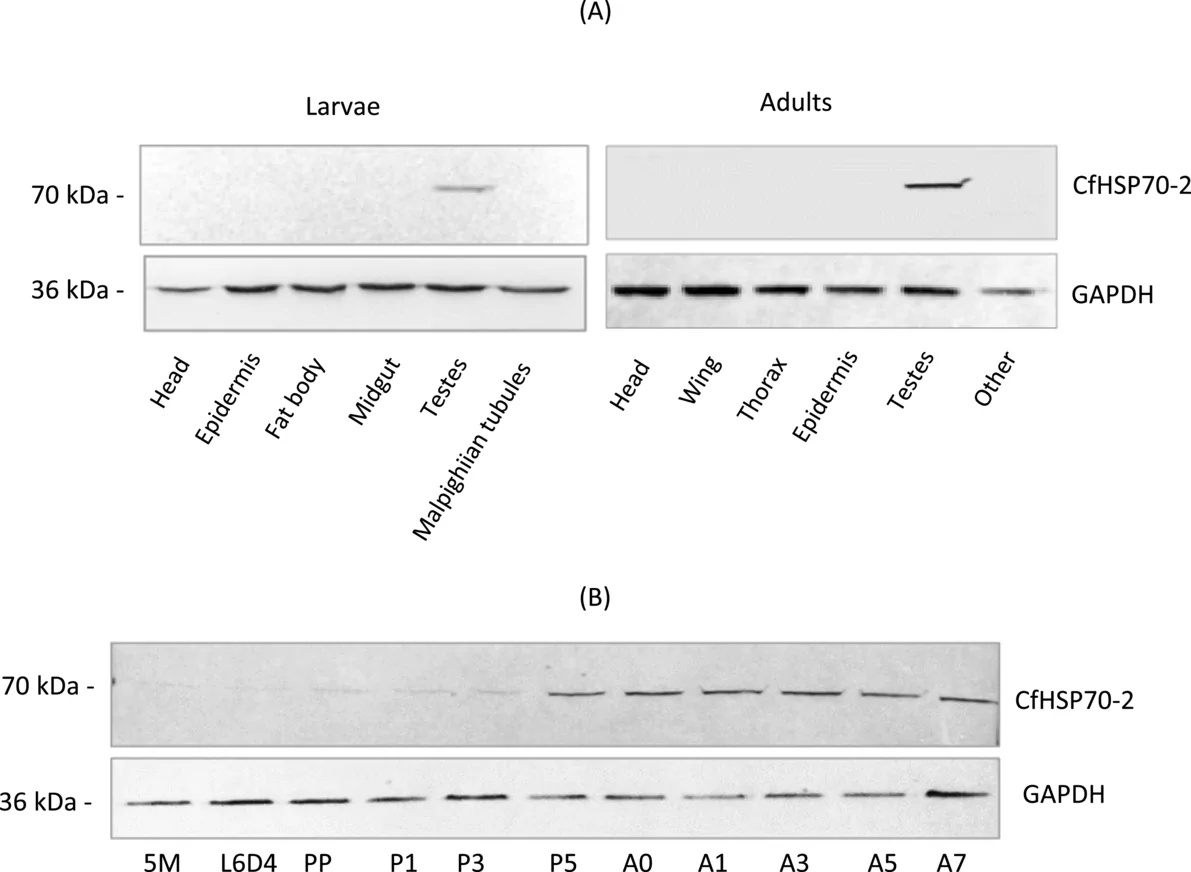
Stage‐ and tissue‐specific expression of CfHSP70‐2 protein. (A) Western blot analysis revealed CfHSP70‐2 protein levels in various tissues, including the head, epidermis, fat body, midgut, testes, and Malpighian tubules, of 4th‐day 6th‐instar larvae, and in the head, wing, thorax, epidermis, and testes of newly emerged adults. (B) CfHSP70‐2 protein expression in testes at different developmental stages. 5 M: 5th‐molting instar larvae; L6D4: 4th‐day 6th‐ instar larvae; PP: pre‐pupae; P1–5: pupae 1 to 5 days following pupation; A0: newly emerged adults; A1–7: adults 1 to 7 days following eclosion. Protein molecular weights are indicated on the left. The expression levels of GAPDH, the loading control, are shown at the bottom.

Western blot analysis confirmed that CfHSP70‐2 protein had a molecular weight of approximately 70 kDa (Figure 2A), it is identical to that calculated from inferred amino acid. Further analysis revealed that CfHSP70‐2 protein was detected in the testes (70 kDa band) at the larval stage, but was absent in all other tissues, including the head, epidermis, fat body, midgut, and Malpighian tubules. Similar results were obtained in the adult stage. Overall, these results indicate that CfHSP70‐2 protein expression is testis‐specific (Figure 2A). In contrast to CfHSP70‐2 protein was nearly undetectable in the testes of 5 M and 6th‐instar larvae, CfHSP70‐2 protein expression gradually increased in the pupa stage, reaching a peak in the late pupa stage and remained elevated during the first 3 days of adulthood. However, CfHSP70‐2 protein expression declined slightly in day‐5 to day‐7 adults.

### 3.3 Effects of Heat Stress, Starvation, Hemin, H_2_O_2_ Treatments on *CfHSP70‐2* Expression

**Figure 3:**
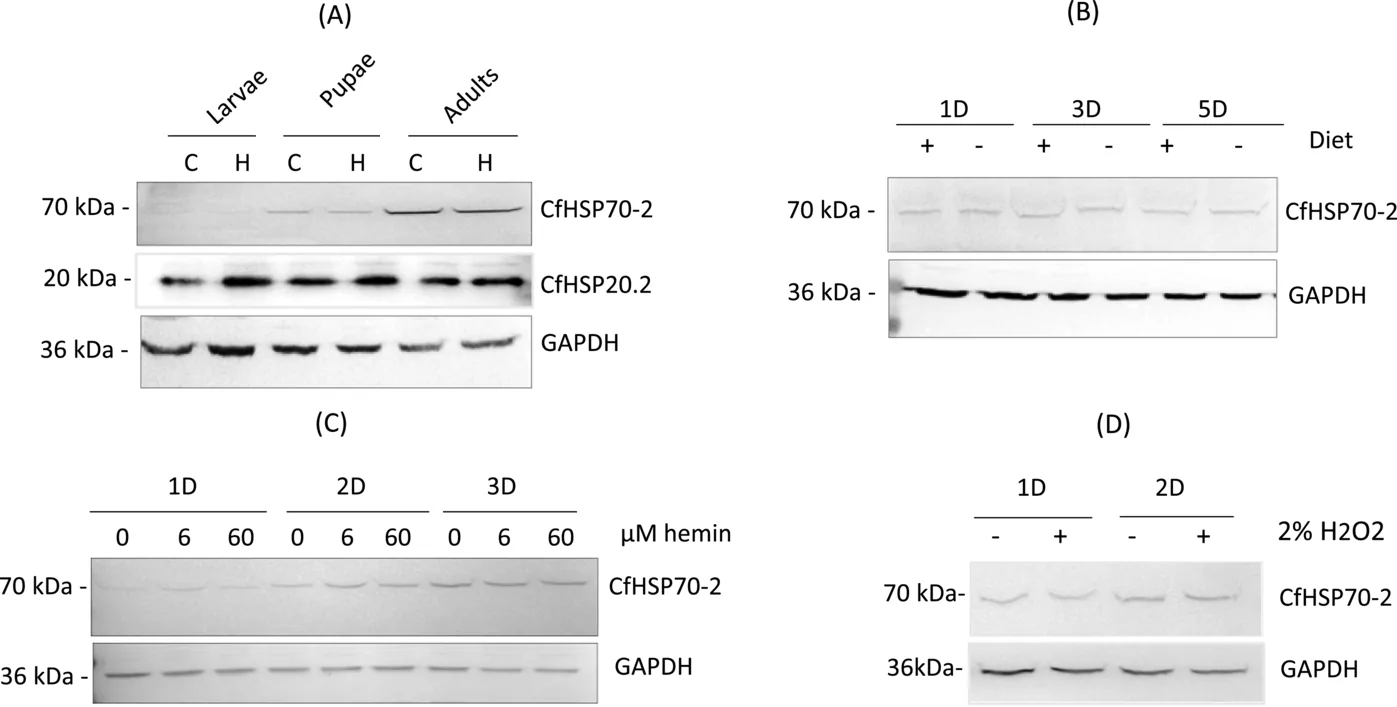
Effects of abiotic stressors, including heat stress, starvation, Hemin, and H_2_O_2_ on CfHSP70‐2 expression. (A) Effect of heat shock treatment on CfHSP70‐2 expression. 4th‐day 6th‐instar larvae (L6D4), 3rd‐day pupae (P3), and newly emerged adults (A0) were heat treated, after which the testes were collected to detect CfHSP70‐2 expression using western blot analysis. CfHSP20.2 was used as a positive control (Quan et al. 2023). (B) Effect of starvation on CfHSP70‐2 expression. 1st‐day 6th‐instar larvae (L6D1) were reared under standard conditions (diet was omitted for experimental groups). Testes were dissected on days 1, 3 and 5 for subsequent analysis. (C) Effect of hemin injection on CfHSP70‐2 expression. Day 1 male pupae were injected with phosphate‐buffered saline (0) or hemin at final concentrations of 6 and 60 µM, after which the testes were collected dissected on day‐1, ‐2, and ‐3 to examine CfHSP70‐2 expression levels. (D) Effect of 2% H_2_O_2_ injection on CfHSP70‐2 expression. Day 1 male pupae were injected with 2% H_2_O_2_ or phosphate‐buffered saline control. Testes were dissected on day‐1 and day‐2 to examine CfHSP70‐2 expression using western blotting. GAPDH was used as a loading control. Each experimental group had four independent replicates.

Previous studies have demonstrated that heat or starvation has little effect on *CfHSP70‐2* mRNA expression (Quan et al. 2023). Therefore, we investigated the effects of heat shock and starvation on the expression of CfHSP70‐2 protein using western blot analysis. As shown in Figure 3A, thermal treatment did not significantly affect CfHSP70‐2 protein expression in the testes at the larval, pupal, and adult stages. In contrast, CfHSP20.2 protein was elevated following 37°C x 1 h heat treatment in the testes at all stages (Figure 3A). To investigate the effects of starvation on CfHSP70‐2 expression, we examined CfHSP70‐2 protein in the testes of L6D1 larvae during starvation. Notably, CfHSP70‐2 protein expression in the larvae was not significantly affected by starvation (Figure 3B). Hemin and H_2_O_2_ can induce stress responses in several cells (Courgeon et al. 1988; Yoshima et al. 1998), to determine whether these responses occur in SBWs, we investigated the effects of hemin or H_2_O_2_ injection on CfHSP70‐2 protein levels in the testes at the pupal stages. Importantly, CfHSP70‐2 protein expression was not significantly affected by hemin or H_2_O_2_ injection following treatments (Figure 3C and D).

### 3.4 Immunolocalization of CfHSP70‐2 in the Testis During Development

**Figure 4:**
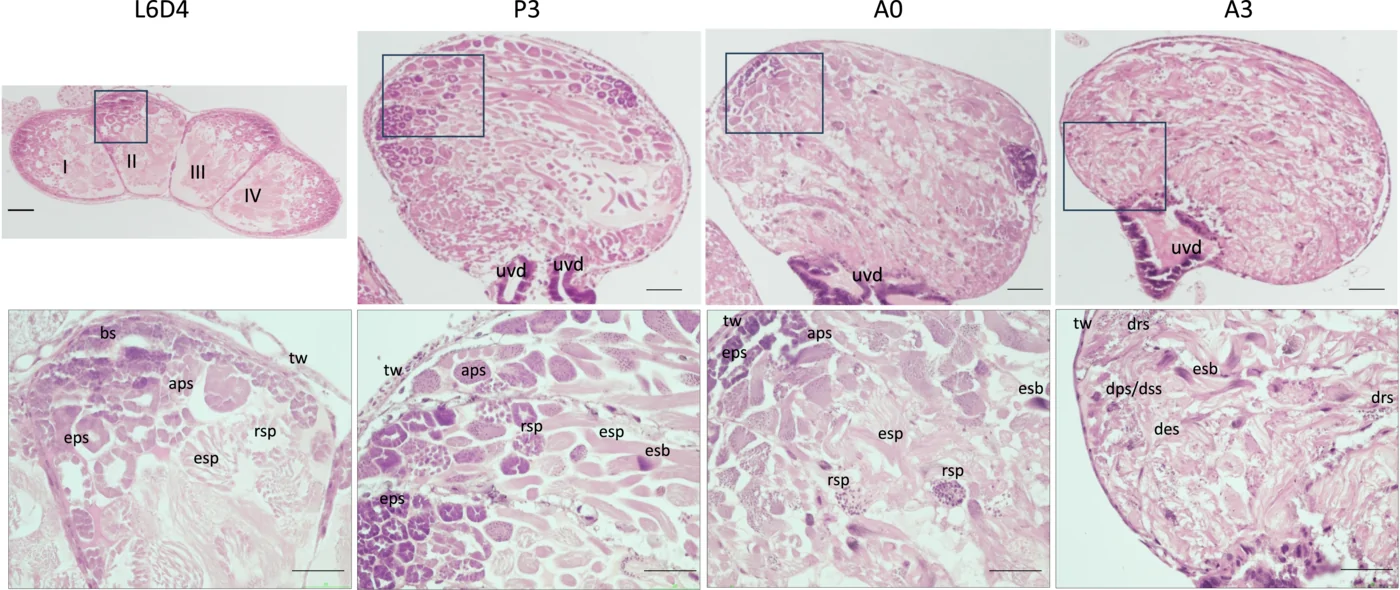
Hematoxylin and eosin (H&E) staining of paraffin sections of testes. Cross‐sectional view of a testis stained with H&E during spermatogenesis in spruce budworm (SBW) at the larval to adult stages. Testes of the 4th‐day 6th‐ instar larvae (L6D4) contained four follicles containing all stages of spermatogenesis. The periphery was covered by the testis wall (tw); inside the follicles, spermatogenesis can be divided into three regions from the apex to center: the growth, maturation/reduction, and transformation zones (Polanska et al. 2005). In the growth zone, spermatogonia divide and increase in size to form spermatocytes, including bipotential spermatocytes (bs), apyrene primary spermatocytes (aps), and eupyrene primrary spermatocytes (eps) which are closely packed in the cysts, and second spermatocytes. In the maturation and reduction zone, each spermatocyte undergoes two meiotic divisions to form spermatids. In the transformation zone, spermatids undergo spermiogenesis, forming mature spermatozoids comprising round spermatids (rsp) and elongated spermatids (esp). P3: 3rd‐day pupal testis. The paired larval testes lobes were fused to a single sphere coalesced with a paired upper vasa deferentia (uvd) at the base of the testes; primary spermatocytes differentiated into apyrene spermatozoa (aps), which usually have granular nuclei in the central part, and eupyrene spermatozoa (eps), which have nuclei at the anterior end; round spermatids (rsp); elongated spermatids (esp), and eupyrene spermatozoid bundles (esb) were observed in the central region. Ao: newly emerged adult testes. The number of spermatogonia decreased and occupied a more peripheral position in most parts of the testis in fully developed spermatozoids, including elongated spermatozoa (esp) and round spermatids (rsp). A3: 3rd‐day adult testes. Degeneration was observed in various germ cells. Degenerated primary spermatocytes (dps) or secondary spermatocytes (dss), elongated spermatozoa (des), and round spermatids (drs) were observed. Scale bar 50 µM (top images) and 25 µM (bottom enlarged images).

Hematoxylin and eosin staining was performed to investigate the immunolocalization of CfHSP70‐2 protein the larval, pupal, and adult testes during the spermatogenesis (Figure 4). At the larval stage, the pair of kidney‐shaped testis had four distinct follicles, with each follicle containing a germinal cyst of all stages, including bipotential spermatocytes, primary and secondary spermatocytes, round spermatids, and elongated spermatids. At the pupal stage, four distinct follicles merged into one testis, two types of sperm, nucleate (eupyrene) and anucleate (apyrene), were observed. In particular, tightly packed apyrene cysts were located at the periphery of the testis, whereas eupyrene were more loosely distributed in the centers of the testis. In addition, both round and elongated spermatocytes were observed in the pupal stages. Moreover, spermatozoid bundles were also observed. For newly emerged adults, the numbers of spermatogonia decreased and were occupying a more peripheral position, and a large number of apyrene spermatocytes appeared. In day‐3 adult testes, fully developed spermatozoids were observed in the testes, with several degenerated, shrunken primary and secondary spermatocytes.

**Figure 5:**
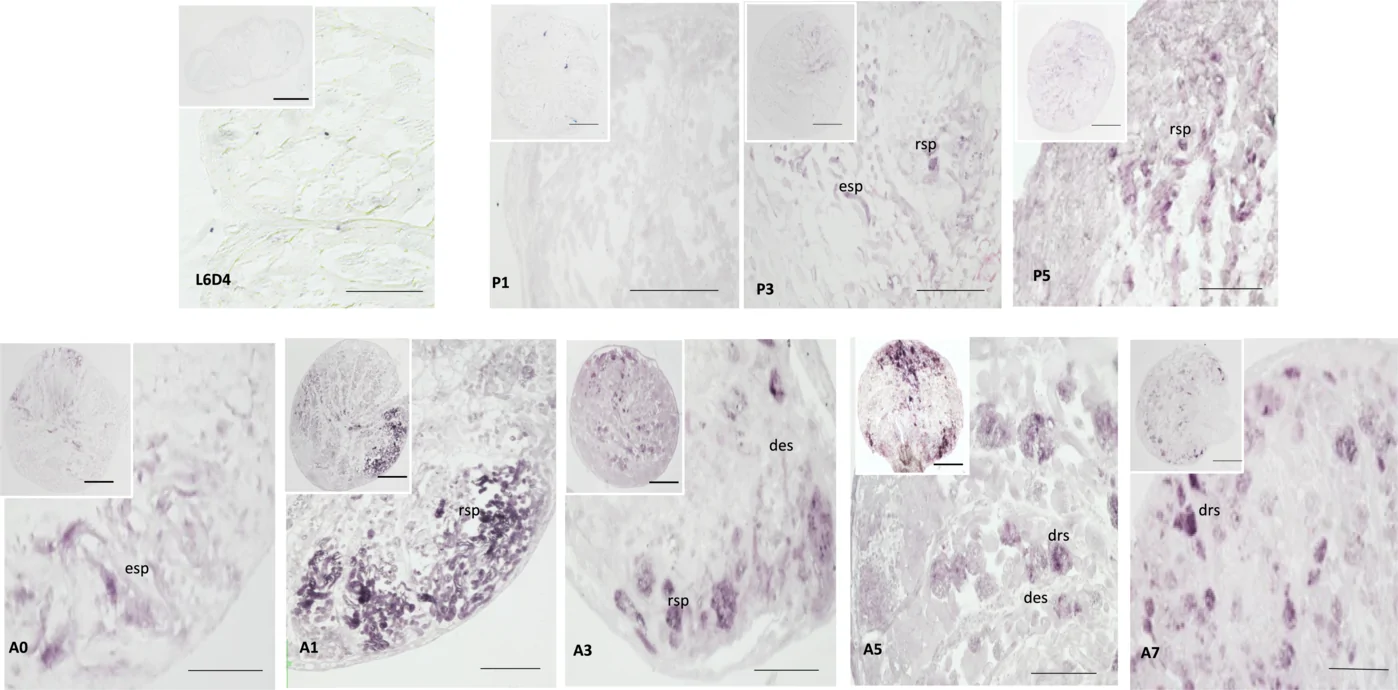
Immunolocalization of CfHSP70‐2 in the testes. Paraffin longitudinal sections of testes from larvae, pupae, and adults were immunologically stained for CfHSP70‐2 with a secondary alkaline phosphatase‐conjugated antibody, followed by incubation with NBT/BCIP. Positive staining displayed brown. L6D4: 4th‐day 6th‐instar larvae; P1‐ 5: pupae 1‐ 5 days after pupation; A0: newly emerged adults; A1‐ 7: adults 1 to 7 days after eclosion. Scale bars 50 µm (insert images) and 10 µm (bottom enlarged images). CfHSP70‐2 signals were very weak or undetectable in the testes of 6th‐instar larvae (L6D4) and early‐stage pupae. For P3 and P5 testes, CfHSP70‐2 signals could be clearly detected in the elongated (esp), round spermatids (rsp), and primary spermatocytes. Strong signals were also detected in P5 testis spermatozoid bundles. CfHSP70‐2 signals were undetected in the testis wall, including the tunica externa and tunica interna at any stage. In A0 testes, CfHSP70‐2 signals were strong in the peripheral region of round and elongating spermatids, whereas the signals were extremely weak in the central region occupied by elongating spermatozoa. A3 testes had similar CfHSP70‐2 distribution patterns, with strong signals detected in the peripheral region, although the signals were mostly observed in round spermatids (elongating spermatids had weak signals). For A5 and A7 testes, strong signals were detected in degenerated round (drs) and elongating spermatids (des), but the latter were weaker.

Immunohistochemical staining was performed on the testes to determine CfHSP70‐2 protein localization and expression patterns (Figure 5). Notably, CfHSP70‐2 signals were nearly undetectable in most L6D4 larvae and P1 testes, except for weak signals in a few spermatogonia near the periphery regions. In contrast, CfHSP70‐2 signals were clearly detected in the round and elongated spermatids in P3 and P5 testes. In addition, a strong signal was detected in spermatozoid bundles in P5 testis. However, CfHSP70‐2 was undetectable in the tunica externa and tunica interna of the testis wall in any stages. In newly emerged adults, the CfHSP70‐2 signals were similar to those detected in P5 testis, although the strongest signals were detected in the apical region of the testis in the spermatozoid bundles and the round spermatids. Slight signals were observed in fully developed mature sperm. Importantly, CfHSP70‐2 signals had similar distribution patterns from A1 to A3 with strong signals detected in the peripheral position, mostly in round spermatids. However, less positive CfHSP70‐2 staining was observed in the elongated mature sperm. In A5 and A7 testes, strong signals were detected in the peripheral position, primarily in degenerated, shrunken round spermatids.

## 4 Discussion

In this study, the ATP‐dependent HSP, CfHSP70‐2, was identified as testis‐specific in SBW with both mRNA and protein detected only in male testes (Figures 1 and 2). Our previous phylogenetic tree analysis identified CfHSP70‐2 homologs in cluster IV in different species (Quan et al. 2020). Recent studies have identified more testis‐specific ATP‐dependent HSP70 genes (Li et al. 2022; Tan et al. 2024; Zhao et al. 2025). Overall, these findings indicate that testis‐specific HSP70s are common in insects and may fulfil certain functions common to various insect species.

In the SBW, DNA and RNA synthesis in the testes increases markedly during the 3rd instar, coinciding with rapid growth and spermatogenesis (Retnakaran 1971). It is also during the period where protein synthesis initiates and continues on through the pupal to early adult stages (Retnakaran 1971). At adult eclosion, 80% of male SBW gametes are mature (Retnakaran 1970). However, further maturation may continue during the post‐eclosion period. Importantly, 2‐ to 4‐day‐old adult males have the greatest mating success, with an insemination rate nearly three times higher than that of 1‐day‐old males (Outram 1971). Although more research is required, the increased expression of CfHSP70‐2, which corresponds to the time when male gametes develop and mate, suggesting that CfHSP70‐2 may play a role in these processes.

In the late adult stages, *CfHSP70‐2* mRNA levels decreased markedly, whereas the protein expression declined only slightly in the testes. Notably, the decrease in *CfHSP70‐2* mRNA levels may indicate weak transcript activity in the testes. However, the decrease in *CfHSP70‐2* mRNA levels coincided with the presence of apoptosis germ cells (Figure 4), indicating a potential correlation. Consistent with CfHSP70‐2 expression, apoptotic germ cells were observed in the testes at the adult stage. Given that certain HSP70s in the HSP70 family exhibit pro‐ or anti‐apoptotic activity, CfHSP70‐2 may have similar functions (Beere 2004; Vydra et al. 2006; Roufayel and Kadry 2019).

In mammals, two members of HSP70 are expressed in male gonads under normal conditions and are thought to be critical for spermatogenesis and male fertility (Radons 2016). In humans and mice, HSP70‐2 is present in the testis, brain, and several other tissues in a cell‐type‐specific manner. For instance, HSP70‐1t is highly expressed in the testis but exhibits low expression in other tissues (Eddy 1999; Daugaard et al. 2007; Scieglinska and Krawczyk 2015). A previous study revealed that HSP70‐2 knockout mice were sterile due to massive germ cell apoptosis (Dix et al. 1996). Recent studies in male *Drosophila* have shown that five members of heat shock cognate 70 (Hsc70) proteins regulate various steps of the insulin signaling pathway, which is essential for spermatocyte growth before male meiosis (Azuma et al. 2021). CfHSP70‐2 expression occurs in later stages than those of HSP70s in mammals and male *Drosophila*. Given that testicular development in SBW primarily occurs during the larval and early pupal stages, CfHSP70‐2 may be involved in sperm maturation and apoptosis.

In mammals, heat shock downregulates HSP70‐2 and Hsc70t expression in the testis, and is partly mediated by the activation of heat shock factor 1 (HSF1) (Widlak et al. 2007; Scieglinska and Krawczyk 2015). In contrast, the present study showed that heat shock did not affect CfHSP70‐2 expression (Figure 3A), indicating that *CfHSP70‐2* is not a heat shock response gene but rather is influenced by internal biological responses. Another factor that adversely affects the SBW population is food shortage. Although the expression levels are low, our early investigation showed that *CfHSP70‐2* mRNA increased somewhat after 3 days starvation. However, we were unable to find any difference in the CfHSP70‐2 protein expression following the starvation of SBW larvae (Figure 3B). These findings may indicate that SBW larval food consumption is not directly correlated with CfHSP70‐2. Hemin can trigger the production of HSPs through a transcriptional process involving heat shock factors (HSFs) (Yoshima et al. 1998). In particular, hemin can activate HSF1, a transcription factor that binds to the promoter region of the gene and triggers HSP70 protein synthesis (Yoshima et al. 1998). H_2_O_2_ is an oxidative stress agent that can activate transcription factors, such as HSF1, or the Janus kinase (JAK)/STAT signaling pathway. Elevated levels of H_2_O_2_ can induce the expression of HSP70, which is a component of the oxidative stress response in cells (Courgeon et al. 1988). Contrary to these findings, present study revealed that neither hemin nor H_2_O_2_ injection into pupae did not alter CfHSP70‐2 protein expression. However, the role of CfHSP70‐2 in oxidative stress remains unknown.

Our study revealed that CfHSP70‐2 was expressed in round spermatids and spermatozoid bundle in adult SBW. Round spermatid are haploid male germ cells containing half the genetic material of a primary spermatocyte. Similar to other insects, SBWs produce two forms of sperm; nucleate (eupyrene), which fertilizes eggs, and anucleate (apyrene) (Retnakaran 1970). Consistent with observations in several lepidopteran species, epyrene sperm appear earlier in the spermatheca than apyrene sperm (Marcotte et al. 2003; Kawamura and Sahara 2002). Accordingly, CfHSP70‐2 was mainly found in apyrene round spermatids. In silkworms, high‐temperature exposure (32°C for 72 h) during the spinning stage induces male sterility (Sugai and Hanaoka 1972; Fugo and Arisawa 1992), which has been attributed to dysfunction of apyrene sperm. Similarly, heat‐shock applied to the male pupae, induced high levels of sterility due to the lack of normal apyrene sperm (Sahara and Kawamura 2002). Apyrene sperm are indispensable in the silkworm fertilization, with polyploid apyrene sperm acting as a substitute for diploid sperm (Sahara and Kawamura 2002). Based on these findings, it can be speculated that CfHSP70‐2 plays a role in protecting round spermatids from high temperatures although further clarification is necessary. Spermatozoid bundles are groups of sperm cells that stick together to form a single unit. This bundling increases the efficiency of sperm transport, offers environmental protection, and aids in competition for fertilization in several insects, including beetles, flies, termites, and ants (Morcillo i Soler et al. 2022; Lupetti et al. 2024). CfHSP70‐2 was detected in the spermatozoid bundles of late pupal and early adult testes (Figure 5), suggesting its role in these functions.

Apoptotic death of spermatogenic cells is common in mammals and is estimated to eliminate up to 75% of mature spermatozoa. Among the various germ cell types, primary spermatocytes are the most sensitive to local heat stress (Lue et al. 1999). Different HSPs can interfere with both anti‐ and pro‐apoptotic pathways (Beere 2004; Roufayel and Kadry 2019). Therefore, CfHSP70‐2 expression in testes suggests a potential involvement high‐temperature‐induced apoptosis of sperm cells.

Recent studies have identified several relationships between HSP genes and male insect fertility. Li et al. (2022) found a testis‐specific HSP, MaltHSP70‐2 in *M. alternatus*, that contributes to thermal resistance in the primary spermatocyte. Tan et al. (2024) linked the HSPs ZcHSC70‐1 and ZcHSC‐2 in *B. cucurbitae* to the regulation of spermatogenesis as they are expressed in the transformation and maturation regions of the testis, and their down‐regulation results in diminished spermatid production and male fertility. Zhao et ai. (2025) identified another testis‐specific HSP, SfHsp68A in *S. frugiperda*, where it was determined that deletion of this gene led to decreased testis size, sperm counts, and egg hatch rates. It is unclear if CfHSP70‐2 plays similar roles to the HSP genes previously mentioned, however, the expression patterns, thermal responsiveness, and location of CfHSP70‐2 in SBW differs from those of the preceding species.

Global climate warming will alter the forest insects' population abundance and distribution, therefore a comprehensive understanding of how extreme heat events will affects testis development and spermatogenesis is critical for determining future patterns of survival, population dynamics, and distribution. Research in the realm of insects is considerably limited in contrast to the vast understanding of heat‐induced fertilization in mammals. Our study offers fundamental insights into the functions and mechanisms of HSP proteins in testis development and spermatogenesis. Further research is required to identify the target proteins and additional testis‐specific genes to elucidate the roles of HSPs in male sterility induced by thermal stress.

## Author Contributions

**Guoxing Quan:** conceptualization, funding acquisition, writing – original draft, validation, supervision, investigation, writing − reviewing and editing. **Hannah Soares:** Investigation, writing − original draft, writing − reviewing and editing, formal analysis.

## Conflicts of Interest

The authors declare no conflicts of interest.

## Supporting information


Supporting File 1

Supporting File 2

## Data Availability

The data that supports the findings of this study are available in the supporting material of this article.
